# Outcome of Santulli enterostomy in patients with immaturity of ganglia: single institutional experience from a case series

**DOI:** 10.1186/s12893-022-01849-9

**Published:** 2022-11-18

**Authors:** Zhixiong Lin, Mingkun Liu, Lei Yan, Lijuan Wu, Jianxi Bai, Dianming Wu, Yifan Fang, Yu Lin

**Affiliations:** 1 Department of Pediatric Surgery, Fujian Maternity and Child Health Hospital, College of Clinical Medicine for Obstetrics & Gynecology and Pediatrics, Fujian Medical University, No. 18 Daoshan Road, Fujian 350001 Fuzhou, China; 2 Department of Pediatric Surgery, Fujian Children’s Hospital (Fujian Branch of Shanghai Children’s Medical Center), College of Clinical Medicine for Obstetrics & Gynecology and Pediatrics, Fujian Medical University, Fuzhou, 350000 Fujian China; 3 Department of Pediatric Surgery, Fujian Branch of Shanghai Children’s Medical Center Affiliated to Shanghai Jiaotong University School of Medicine, Fuzhou, 350000 Fujian China

**Keywords:** Santulli enterostomy, Immaturity of ganglia, Allied disorders of Hirschsprung’s disease, Barium enema

## Abstract

**Background:**

Immaturity of ganglia (IG) is an extremely rare disease and always requires surgical intervention in the neonatal period, but without guidelines to choose the ideal enterostomy procedure, the timing of stoma closure remains controversial. The aim of this study was to report our experience using Santulli enterostomy for the treatment of nine infants diagnosed with IG.

**Methods:**

Patients who underwent Santulli enterostomy and were diagnosed with IG in our center between 2016 and 2021 were retrospectively studied. Temporary stoma occlusion and a 24-h delayed film of barium enema (BE) were performed to evaluate intestinal peristalsis function to determine the timing of stoma closure. The demographic data, clinical and radiological findings, stoma occlusion and stoma closure results were explored.

**Results:**

A total of 9 infants underwent Santulli enterostomy and were diagnosed with IG postoperatively. Their median gestational age at birth was 36 weeks (range 31–42), and their median birth weight was 2765 g (range 1300–3400). All patients had symptom onset in the neonatal period, including abdominal distension and biliary vomiting. Eight patients showed obvious small bowel dilatation in the plain films, except for one patient’s films that suggested gastrointestinal perforation with free gas downstream of the diaphragm. BE was performed in 6 patients, all of which had microcolons. The median age at operation was 3 days (range 1–23). Seven patients had an obvious transitional zone (TZ) during laparotomy, and the position of the TZ was 25–100 cm proximal above the ileocecal (IC) valve. Immature ganglion cells were present in the colon in 7 patients and the terminal ileum in 6 patients. The median age of successful stoma occlusion was 5 M (range 2–17) and 8 M (range 4–22) at ostomy closure. There was little or no barium residue in the 24-h delayed film of BE before stoma closure, and all patients were free of constipation symptoms during the follow-up.

**Conclusion:**

Santulli enterostomy appears to be a suitable and efficient procedure for IG, combined with temporary stoma occlusion and 24-h delayed film of BE to evaluate the recovery of intestinal peristalsis function.

## Introduction

Immaturity of ganglia (IG) is an extremely rare disorder characterized by small and extremely immature ganglion cells in both the nucleus and cell in the intestinal wall, and is a kind of allied disorder of Hirschsprung’s disease (ADHD) [1]. This leads to intestinal peristalsis dysfunction and causes low gut obstruction. Most infants experience onset in the neonatal period and need surgical intervention when conservative therapy is ineffective. Enterostomy is the main surgical treatment for infants with IG, including double-barreled ileostomies and loop ileostomy [2–4]. Currently, there are no clear guidelines for the choice of enterostomy type.

Santulli enterostomy is an ostomy in continuity procedure that enables rapid decompression of the proximal jejunum and recovery of intestinal continuity. The procedure has been successfully applied in the treatment of multiple intestinal atresia (MIA) and necrotizing enterocolitis (NEC); it can prevent postoperative short bowel syndrome (SBS) and shorten the duration of parenteral nutrition (PN) therapy [5, 6]. At present, there is no report on the application of Santulli enterostomy in IG. Therefore, the aim of this study was to summarize the outcomes and experiences of Santulli enterostomy in IG.

## Material and methods

### Patient samples

Inpatient records were retrospectively reviewed to identify children who underwent Santulli enterostomy procedures between June 2016 and June 2021 and had a pathological report of immature ganglion cells in the intestinal wall. All the patients presented with low intestinal obstruction, extreme abdominal distension or bilious vomiting. All patients accepted conventional therapy including fasting, gastrointestinal decompression via the stomach and anal tubes, the use of laxatives and enemas, anti-infection therapy and nutritional support. Laparotomy and Santulli enterostomy were performed after conservative treatment was ineffective or if there was a suspicion of intestinal atresia, total colonic aganglionosis (TCA) and perforation. When the presence of ganglion cells was proven by immediate frozen pathological examination during the operation, multiple full-thickness biopsies of the colon and ileum were taken for ADHD was suspected. Patients who received Santulli enterostomy and whose postoperative pathology from the permanent section report showed immature ganglion cells in the bowel wall were included in this study. Patients who were diagnosed with anorectal malformation, Hirschsprung’s disease (HD), other types of ADHD, or meconium ileus syndrome and those lost to follow-up were excluded. The stoma was temporarily occluded with a latex Foley balloon catheter starting at one month after Santulli enterostomy. The time to close the stoma is influenced by evidence of the recovery of intestinal peristalsis function. Demographic data, clinical presentations, radiological findings, operative procedures, stoma occlusion and stoma closure results were collected in this study. The study began after approval by the ethics committee at Fujian Maternity and Child Health Hospital, College of Clinical Medicine for Obstetrics & Gynecology and Pediatrics, Fujian Medical University (No. 2020YJ177).

### Diagnosis of IG

The diagnosis of IG was made by histopathology diagnosis according to the criteria recommended by the Japanese Study Group of ADHD in 2015 [1]. The postoperative pathological report showed a normal number and distribution of ganglion cells in the intestinal wall, but the cell were small and extremely immature in both the nucleus and cells.

### Temporary stoma occlusion

Temporary stoma occlusion was started one month after enterostomy if the stoma was free of complications, such as necrosis, prolapse, retraction, and stenosis. Occlusion was characterized as either half-occlusion or complete occlusion.

**Fig. 1:**
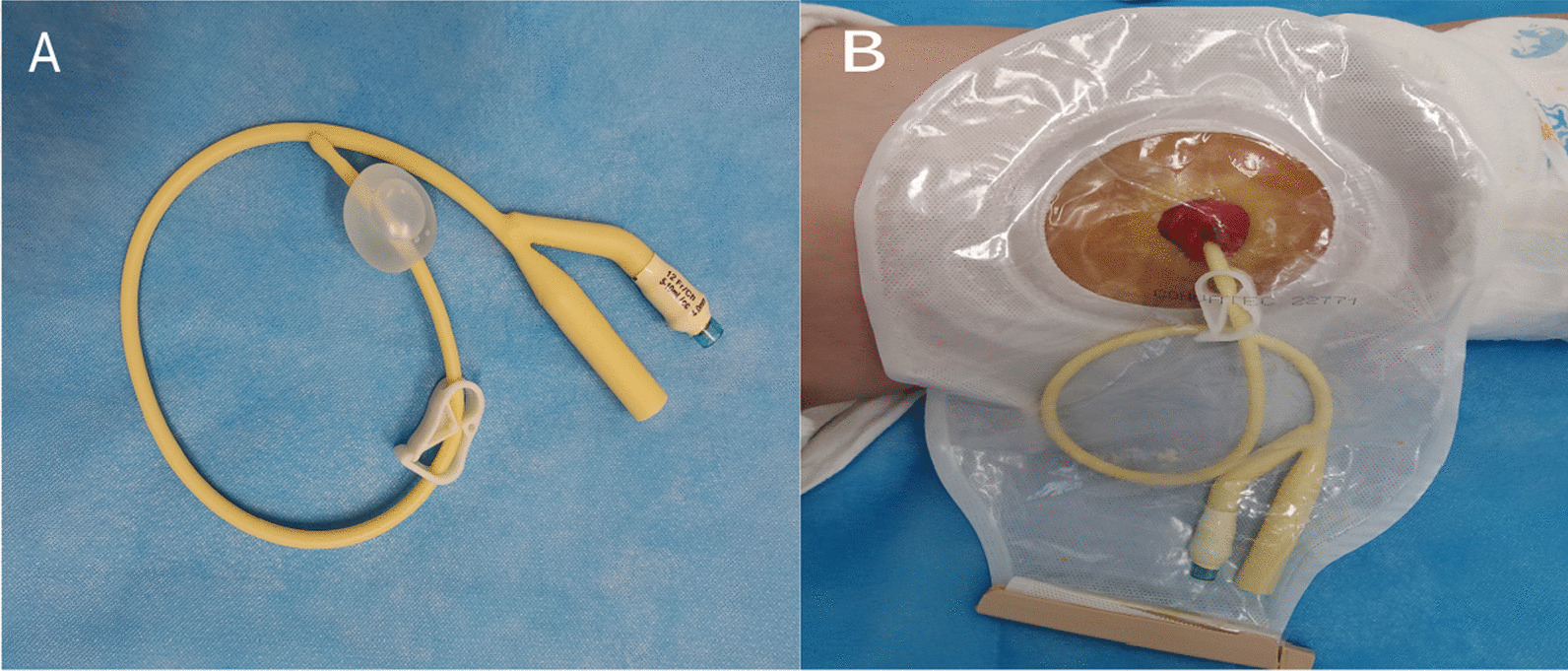
Temporary stoma occlusion and materials. **A** A latex Foley balloon catheter with a clip; **B** The stoma was occluided with the Foley catheter

Half-occlusion was achieved by inserting a Foley catheter (Fig. 1A) approximately 5 cm into the enterostomy, injecting 3 mL saline into the aerocyst and closing the catheter with a clip. The Foley catheter was moved slightly outward to place the stoma in the half-closed state, and thus some of the chyme from the proximal intestine could pass into the distal bowel. However, if the patient tolerated the procedure for half-occlusion without obvious discomfort, the procedure to achieve complete occlusion was implemented one month later.

Complete occlusion was achieved byinjecting 5–7 mL saline into the aerocyst, closing the catheter with a clip, and moving the Foley catheter outward to ensure that the intestinal outlet was completely closed (Fig. 1B). Therefore, the proximal intestinal contents will completely pass through the anastomosis into the distal bowel. During the procedure, the color of the stoma mucosa was observed closely for 5–10 min. If the stoma mucosa became blackened, the balloon was released quickly to decrease the pressure on the intestinal wall.

If frequent crying, poor appetite, abdominal distension, vomiting, constipation or gastrointestinal bleeding (melena, bloody stool) occurred after the stoma was completely occluded, the Foley catheter was removed and then one month later, the procedure was tried again. The occlusion was successful if patient did not show any of the above symptoms during stoma occlusion. After successful occlusion for 72 h, the Foley catheter was removed for 24 h avoid obstructing the blood supply at the stoma caused by long-term compression.

### The timing of stoma closure

**Fig. 2:**
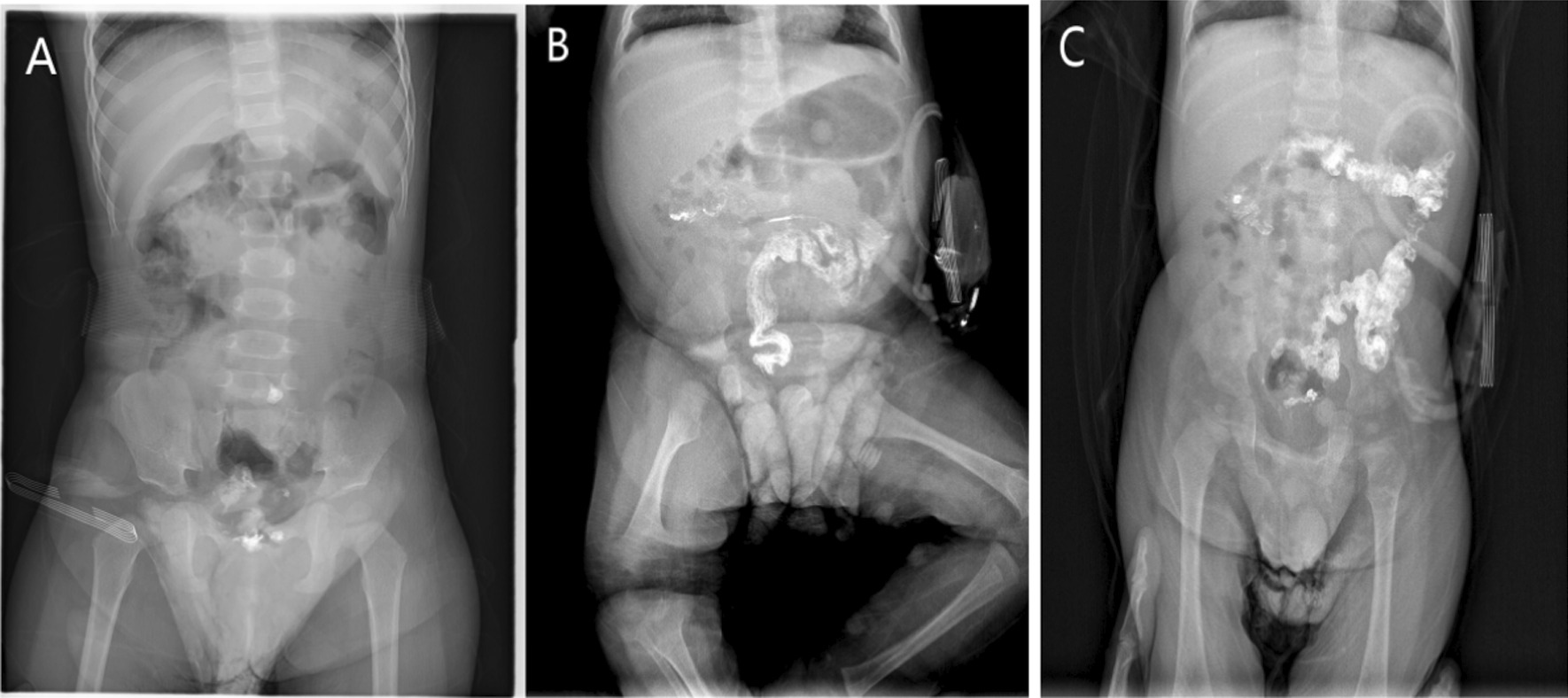
The different degrees of barium residue in 24-h delayed film of barium enema (BE) after the stoma successful occlusion. **A** Small amount of barium residue, **B** Moderate amount of barium residue, **C** Large amount of barium residue

Successful occlusion indicated substantial recovery of intestinal peristalsis function. Then, a 24-h delayed film of barium enema (BE) was performed to further confirm the colonic motion function [7]. The ileostomy was closed after the contrast medium was completely excreted or all but a small amount of residue (barium residue in the rectum, see Fig. 2A) was removed. However, when barium evacuation showed a moderate amount (barium residue in the rectum and sigmoid colon, see Fig. 2B) or a large amount of barium residue (barium residue from anus to the level of descending colon, or even into the transverse colon., see Fig. 2C), the entire occlusion and testing procedure was repeated at least a month later.

### Statistical analysis

Data were analyzed using SPSS software (IBM SPSS Statistics for Windows, version 21.0). Descriptive statistics were used for this study. The median was used for variables with a skewed distribution. Enumeration data are expressed as case numbers and percentages (%).

## Results

From June 2016 to June 2021, a total of 9 infants (7 boys and 2 girls) with a postoperative pathological diagnosis of IG who underwent Santulli enterostomy were included in this study. There were 6 premature infants and 3 term infants. They were born at gestational ages ranging from 31 to 42 weeks, and their median birth weight (BW) was 2765 g (range 1300–3400). All children (100%) had symptom onset in the neonatal period, including abdominal distension and biliary vomiting. Delayed passage of meconium occurred in 5 patients (56%), and respiratory failure occurred in 3 patients (33%). In the abdominal plain films of 9 children, 1 patient (case No. 9) had free gas downstream of the diaphragm, suggesting gastrointestinal perforation. The other 8 patients showed obvious dilatation of the small intestine, of which 2 patients (cases No. 1 and 7) were accompanied by obvious gas–liquid levels. BE was performed in 6 patients (67%), all of which showed microcolons. Patient information is summarized in Table 1.

**Table 1:** Demographic data, clinical presentations and radiological findings

Case no.	GA (weeks)	BW (grams)	Time of conservative therapy (days)	Symptomatology	Plain abdomen	BE
1 M	35	2765	1	Marked abdominal distension, bilious vomiting, respiratory failure	Marked small bowel dilatation with air fluid level	Microcolon
2 M	36	1620	3	Delayed passage of meconium, abdominal distension, bilious vomiting	Generalized small bowel dilatation	Microcolon
3 M	33	2270	4	Delayed passage of meconium, abdominal distension, bilious vomiting,	Generalized small bowel dilatation	Microcolon
4 M	39	3400	3	Delayed passage of meconium, abdominal distension, bilious vomiting	Marked small bowel dilatation	Not done
5F	42	3270	2	Delayed passage of meconium, abdominal distension, bilious vomiting	Marked small bowel dilatation	Microcolon
6 M	36	3000	4	Abdominal distension, bilious vomiting	Marked small bowel dilatation	Not done
7 M	37	3100	3	Delayed passage of meconium, abdominal distension, bilious vomiting	Marked small bowel dilatation with air fluid level	Microcolon
8F	31	1300	10	Abdominal distension, bilious vomiting, respiratory failure	Generalized small bowel dilatation	Microcolon
9 M	33	1980	1	Marked abdominal distension, peritonitis, bilious vomiting, clinical sepsis	freeing gas under diaphragm	Not done

*GA* gestational age, *BW* birth weight, *BE* barium enema

The median age at operation was 3 days (range 1–23). Three of these patients (Nos. 1, 5, 9) underwent laparotomy one day after birth for gastrointestinal perforation, a high suspicion of intestinal atresia and total colonic aganglionosis, and the other 6 patients (67%) underwent surgical treatment after conservative treatment was ineffective. Of the 9 patients, 7 (78%) had an obvious transitional zone (TZ) during the operation. The position of the TZ was 25–100 cm proximal above the ileocecal (IC) valve. One patient (case No. 5) had ileal atresia (type Шa) at a distance of 30 cm above the IC valve, and one case (case No. 6) had intestinal adhesion, torsion and necrosis 35 cm above the IC valve. Multiple full-thickness biopsies of the colon and ileum were taken, and the surgeon decided to perform Santulli enterostomy based on the condition of the bowel during laparotomy. The enterostomy position range was 10–100 cm proximal above the IC valve.

Postoperative pathology of all patients showed the presence of immature ganglion cells in the intestinal wall, immature ganglion cells were found in the colon in 7 patients (78%), in the terminal ileum in six patients (67%). The operative findings and postoperative pathology are described in Table 2.

**Table 2:** Operative findings and operative procedures

Case no.	Age at operation (days)	Operative findings	Site of Santulli enterostomy (distal from the IC valve)	Pathological reports (permanent section)
1 M	1	Tz at the terminal ileum 40 cm above the IC valve; Microcolon	40 cm	Terminal ileum at the Tz, terminal ileum, transverse colon, sigmoid colon—immature ganglion cells
2 M	3	Tz at the terminal ileum 85 cm above the IC valve; Microcolon	85 cm	Terminal ileum, transverse colon, sigmoid colon and rectum—immature ganglion cells
3 M	4	Tz at the terminal ileum 100 cm above the IC valve; Microcolon	100 cm	Terminal ileum at the Tz—normal ganglion cells; Transverse colon, sigmoid colon —immature ganglion cells
4 M	3	Tz at the terminal ileum 50 cm above the IC valve; Microcolon	50 cm	colon—normal ganglion cells; Terminal ileum —immature ganglion cells
5F	1	Type Шa intestinal atresia 30 cm above the IC valve; Microcolon	30 cm	Terminal ileum—immature ganglion cells
6 M	13	35 cm ileum above the IC valve had adhesion, torsion and necrosis; Microcolon	10 cm, the necrotic intestinal were removed and an Santulli enterostomy was performed	Transverse colon, rectum—numerous immature ganglion cells; Terminal ileum—normal ganglion cells
7 M	3	Tz at the terminal ileum 25 cm above the IC valve; Microcolon	25 cm	Terminal ileum, transverse colon, rectum—immature ganglion cells
8F	23	Tz at the terminal ileum 30 cm above the IC valve; Microcolon	50 cm	Terminal ileum, transverse colon—normal ganglion cells; Rectum—immature ganglion cells
9 M	1	Tz at the terminal ileum 30 cm above the IC valve and with intestinal perforation; Microcolon	30 cm	Terminal ileum—normal ganglion cells; Transverse colon, Sigmoid colon—immature ganglion cells

*TZ* transitional zone, *IC* ileocecal

All patients without stomal-related complications 1 month after Santulli enterostomy underwent temporary stoma occlusion testing. One patient (case no. 8) showed good tolerance to stoma closure after the first temporary stoma occlusion at the age of 2 months, and the stoma was closed when the body weight reached 5000 g. The other children had different intolerance symptoms, such as frequent crying, poor appetite, abdominal distension, vomiting, constipation or gastrointestinal bleeding. The median age of successful stoma occlusion was 5 months (range 2–17 months). In the 24-h delayed film of BE before stoma closure, there were 5 patients without barium residue and 4 patients with a small amount of barium residue. The median time of ileostomy closure was 8 M (range 4–22), and the weight ranged from 5000 to 11,000 g. The result of temporary stoma occlusion and 24-h delayed film and time of stoma closure are described in Table 3. All the patients showed no signs of discomfort such as abdominal distension and constipation, and had an excellent prognosis during the follow-up.

**Table 3:** The result of temporary stoma occlusion and 24-h delayed film

Case no.	Age at stoma successful occlusion (months)	Barium residue before stoma closure	BW before stoma closure (g)	Age at stoma closure (months)
1 M	5	Little residue	6500	8
2 M	9	No residue	5400	12
3 M	5	No residue	6500	7
4 M	10	Little residue	11,000	14
5F	5	No residue	6000	7
6 M	4	No residue	8060	7
7 M	17	No residue	10,600	22
8F	2	Little residue	5000	4
9 M	7	Little residue	5200	9

*BW* body weight

## Discussion

IG is a rare disease with an incidence of approximately 1–2 patients per million live births according to a survey in Japan [1]. Pathological biopsy shows that immature ganglion cells in the intestinal wall are the main feature of IG, resulting in neonatal functional obstruction, especially in preterm infants. Leiri [1] summarized five characteristics of the clinical diagnosis of IG through a 10-year survey in Japan: neonatal intestinal obstruction, lesion extending to small bowel involvement, symptom improvement with age, microcolon or narrowing of the left colon, and caliber change on laparotomy. Although most of the patients in this study had highly consistent manifestations, full-thickness pathological biopsy is still regarded as the gold standard for the diagnosis of IG [8].

Animal and human studies have shown that immature ganglion cells can gradually mature with time, which is a normal physiological process [2, 9–11]. Several researchers have suggested that this is a self-limiting disease and should be managed with conservative treatment without surgical intervention [4]. Miyahara [12] and Venugopal [13] believe that ganglion cells begin to mature one year after birth, and Feichter et al. [14] thought they are not fully mature until 4 years of age. Therefore, the time of complete maturation of ganglion cells is unclear, and the optimal length of conservative treatment cannot be determined. In our case, all children successfully underwent stoma closure within 22 M with a favorable prognosis. Therefore, we believe that ganglion cells can fully develop and mature before 2 years of age.

Although conservative treatment can be applied to give the ganglion cells time to mature, many children often need surgical intervention because of systemic deterioration, intestinal perforation and suspected gastrointestinal malformation [1, 2, 15]. Enterostomy is the main surgical intervention. In Japan, 84.6% of IG patients undergo double-barreled ileostomies [1], and loop ileostomy is another choice [3], but in our institution, we use Santulli enterostomy. Santulli enterostomy is an ostomy in continuity with a side-to-end anastomosis first proposed by Santulli and Blanc [16] in 1961 for the treatment of MIA. The chyme of the proximal intestine can pass into the distal bowel to avoid SBS and reduce the duration of PN therapy [5, 17]. IG patients also face similar problems with less intestine to absorb nutrition as the ostomy position moves forward. Different from Bishop-Koop enterostomy (another continuous ostomy with end-to-side anastomosis), Santulli enterostomy can eliminate intestinal obstruction, allowing complete decompression of the proximal jejunum, which is also a reason why we chose to perform Santulli enterostomy in IG patients.

With the maturation of ganglion cells in the intestinal wall, when is the best time to close the ileostomy? Holschneider et al. [18] recommend rebiopsy to identify the maturation of the intramural plexus 12–18 months after the initial intestinal wall histological biopsies. Recently, the studies by Burki [2] and Niramis [3] showed that the ganglion cells in the intestinal wall were mature, as proven by a rectal rebiopsy after 3 months, and this is, therefore, a suitable time to close the enterostomy. However, in Burki’s study, although mature ganglion cells were confirmed by rectal biopsy in patients No. 2 and No. 7, their symptoms did not improve immediately. The constipation symptoms in patient No. 2 did not disappear until 3 weeks later. Patient No. 7 still suffered from persistent constipation until the age of 2. Therefore, the maturation of ganglion cells does not mean the complete recovery of intestinal function and the improvement of children's symptoms, and whether this is the best time to close the stoma is debatable. Moreover, a full-thickness rectal biopsy can only obtain a small quantity of tissue and requires general anesthesia, and it is associated with some morbidities, including perforation, bleeding, and infection [19, 20].

In our study, we simulated the state of stoma closure by temporary stoma occlusion, which is simple, reversible and without surgical intervention [16]. It can verify the recovery of intestinal peristalsis function and restore intestinal absorption, facilitate maturation of distal intestinal function and improve the nutritional status of children. Yoo [7] showed that a 24-h delayed film of BE is a supplementary method for evaluating colonic motility in children younger than 4 years. Therefore, before closing the stoma, we applied a 24-h delayed film of BE to verify intestinal peristalsis function. In our experience, the ileostomy can be closed after the contrast medium is completely excreted or there is only a small amount of residue after successful occlusion of the stoma. In this study, all the patients with IG successfully underwent stoma closure and they were free of constipation symptoms during a maximum follow-up of 5 years.

As early as 1961, Santulli proposed a method of temporarily occluding the stoma with Pott's type of clamp on the fifth day after Santulli enterostomy [16], but there have been very few reports since the original study. Santulli enterostomy also has obvious advantages in stoma takedown [21]. It is simple and requires very little additional resection of the intestine [22] and can even be performed under local anesthesia [23]. Recently, Pan et al. [24] applied T-type enterostomy in infantile gastrointestinal diseases. Ihis showed that T-type enterostomy can improve intestinal function and nutrient status through stoma occlusion before enterotomy closure, which was similar to the methods adopted in this study. In this study, we applied Santulli enterostomy in patients with IG, combined with temporary stoma occlusion and 24-h delayed film of BE. The median time of ileostomy closure was 8 M, which is slightly later than the 3.5–7 M previously reported by Niramis [3]. However, our method does not require a second full-thickness colorectal biopsy, reducing harm to the children and achieving the same favorable prognosis.

Of course, there were a few limitations in our study. This single-center study included a small sample of patients and had no control group; the low incidence of the disease contributed to the small sample size. The procedure to obtain a 24-h delayed film increases the radiation exposure to pediatric patients, but compared with the injury caused by full-thickness colorectal biopsy, it is worth it.

## Conclusion

Santulli enterostomy appears to be a suitable and efficient procedure for IG, combined with temporary stoma occlusion and 24-h delayed film of BE to evaluate the recovery of intestinal peristalsis function, and the stoma can be successfully closed. Considering the limitations of this study, the role of Santulli enterostomy combined with temporary stoma occlusion needs to be confirmed in a future prospective study.

## Data Availability

The datasets used and/or analyzed during the current study are available from the corresponding author (Y Lin; ly2018202110@163.com) on reasonable request.

## Abbreviations

IG: Immaturity of ganglia
BE: Barium enema
TZ: Transitional zone
IC: Ileocecal
ADHD: Allied disorder of Hirschsprung’s disease
MIA: Multiple intestinal atresia
NEC: Necrotizing enterocolitis
PN: Parenteral nutrition
TCA: Total colonic aganglionosis
HD: Hirschsprung’s disease
BW: Birth weight
